# Ovariotomy during Pregnancy

**Published:** 1891-10

**Authors:** Christian Fenger

**Affiliations:** Chicago, Ill.


### OVARIOTOMY DURING PREGNANCY.*

BY CHRISTIAN FENGER, M. D., CHICAGO, ILL.

It would seem strange to bring this important subject before the Society with only one case as an illustration. I do not pretend to bring forward anything new or anything of my own, in this connection, but merely desire to present to the Society the thoughts and reflections that I experienced after looking over the literature on the subject. This has been the more interesting to me because of the radical changes in the views as to the

*Read before the Chicago Gynecological Society, May 22d, 1891.

choice of treatment of this condition which have taken place within the last ten years.

CASE.—Mrs. G. E., 30 years of age, primipara. Health always good up to the time of this sickness; she had never been treated for any uterine disease. First menstruated at 14; until the nineteenth year she was occasionally troubled with frequent and profuse menstruation. From the nineteenth to the twentieth year the menstrual flow was regular, but scanty. After the twentieth year it again became normal, and continued so until the time of the last menstruation, May 21st, 1890.

She was married in 1886, at the age of 25, and was well from that time until pregnancy, with the exception of some attacks of pain in the lower part of the abdomen, radiating from the lumbar to the inguinal regions. The pain would come on suddenly, had no connection with menstruation, would last from fifteen minutes to half an hour, and would be followed for several days by tenderness over the lower part of the abdomen. She generally felt chilly during these attacks, but had neither fever nor vomiting. She has had five attacks in all; the first one five years ago, the second a few days later, the third a month later, the fourth a year after the third, and the last attack during January, 1890. Dr. Hartman, her family physician, to whom I am indebted for the information as to her previous history, considered these attacks to be ovarian colic. She consulted Dr. Hartman on July 26th, 1890, when she complained of failing health, general weakness, loss of appetite and flesh, having lost sixteen pounds within five weeks. She further complained of pain and considerable tenderness in the left inguinal region, and had not menstruated since May 21st.

On bimanual examination the uterus was found slightly enlarged, mobile, and pushed over to the left side by a tumor which partially filled the pelvis minor. It did not appear to be firmly adherent to the uterus. An upper portion of the tumor projected above the brim of the pelvis in the right lower part of the hypogastric region. It appeared movable. The surface, although smooth, was not uniform in appearance, inasmuch as the portion in the large pelvis appeared to be solid, while the portion felt through the vagina was elastic and appeared to fluctuate. Dr. Hartman made a diagnosis of dermoid cyst, and this diagnosis was confirmed by the examination October 24th, 1890. The gravid uterus was now found projecting in the hypogastric region, the size of the uterus of the fourth month. Auscultation revealed uterine bruit, but no fetal heart sounds. The tumor had also increased in size, and on examination was found to almost fill the pelvis minor. The wall was in some parts hard and nodular. The upper part could now be only indistinctly felt, as it was covered by the gravid uterus. The patient had not felt any fetal movements, but had had frequent shooting pains in the mammæ, which as yet were not enlarged or changed in appearance. Her general health had improved during the summer.

October 24th I examined the patient in consultation with Drs. Hartman and Lee, and confirmed the diagnosis of dermoid cyst in the small pelvis on the right side of the uterus, and pregnancy of the fourth month. The ovarian tumor was immovably fixed in the small pelvis, and the vaginal portion of the uterus could now be felt high up to the left side and apparently movable against the tumor.

In consultation held as to what course to pursue, it was thought likely that this ovarian cyst, which almost filled and was incarcerated in the small pelvis, might be a dangerous complication to the delivery, or might rupture later on in the course of pregnancy. After considering the choice between the induction of premature labor and subsequent ovariotomy on the one hand, and ovariotomy during pregnancy on the other hand, the latter was decided upon, and the patient taken to the Emergency Hospital and prepared for laparotomy in the usual manner.

October 30th, in the presence of the doctors from the Polyclinic and some of my students from the college, and assisted by Drs. Bernauer, Lee, and Hartman, the anæsthetic being administered by Dr. Rosa Engert, the operation was performed as follows:

An incision was made in the median line from the symphysis pubis to the umbilicus, the pyriformis muscles transversely divided, and the parietal peritoneum sutured to the skin. The gravid uterus presented through the abdominal wound, and the tumor could be felt deep down and behind the uterus, but was inaccessible until the incision had been prolonged above the umbilicus to midway between the latter and the ensiform cartilage.

On introduction of the left hand into the abdominal cavity a cyst could now be felt of the size of a small child's head, the lower part of the tumor filling the small pelvis to the right of and behind the uterus, an upper portion projecting up into the pelvis major. The cyst was so firmly incarcerated in the small pelvis that it could not be removed so as to bring it up into the wound. As I expected to find a dermoid cyst, I did not want to empty its contents in order to facilitate its removal. Therefore I enlarged the abdominal incision still a little further upward, and everted the gravid uterus out through the wound. The uterus was wrapped in warm aseptic cloths soaked in sterilized water, and was held on the outside of the abdominal cavity and to its left side by Dr. Bernauer.

I now introduced the left hand down into the small pelvis behind the cyst, and lifted it up and out through the abdominal wound. It was found to have a smooth surface and to be non-adherent. After having packed the abdominal cavity around the pedicle, the cyst was removed entire. It was somewhat difficult to ligate the broad ligament, as the pedicle was short, especially in the upper part of the broad ligament, which was unfolded and filled by the gravid uterus. The pedicle was transfixed and then dropped, without, as I usually do, dividing it on the clamp by Paquelin's cautery, because the pedicle was too short to permit the application of the clamp. After dropping the pedicle the cloths around the uterus were removed, and, after turning the patient on the side, a pitcher of sterilized water was poured over the uterus, which, after the removal of the large flat sponges, was replaced. It was somewhat difficult to push the uterus back through the wound, the borders of which had to be tightly drawn during its replacement. Several small, subserous ecchymoses had formed on the surface of the uterus during its stay outside. Small sponges on sponge holders, pushed down behind the uterus, showed the abdominal cavity to be free from blood and serous fluid. The abdominal wound was then united with alternate deep and superficial sutures ; no drainage.

At the end of the operation, which lasted an hour and a quarter, the patient was in natural condition ; pulse 90, strong ; no symptoms of collapse.

The second evening after the operation temperature rose to 100.8°, pulse to 96. During the rest of the first week after the operation the morning temperature did not reach 99°, the evening temperature being about 99°. From the beginning of the third week the temperature remained normal.

During the first two weeks the only important symptom was occasional severe paroxysmal pain, simulating uterine contractions ; it could, however, be controlled by repeated hypodermic injections of a quarter of a grain of morphine. This pain made me fear impending abortion, but it gradually decreased, and entirely ceased at the beginning of the third week.

On the fifth day the dressings were changed and the wound found to be perfectly dry and aseptic. The patient was sitting up at the end of the third week.

The subsequent course of the pregnancy was entirely normal, and on February 19th, 1891, the patient fell in labor, which lasted fifteen hours, the child being delivered by forceps. The child was fully developed, at full term, and weighed six pounds. The convalescence after delivery was not attended by fever, but was somewhat tedious. The patient had only a small quantity of milk, and so after three weeks artificial alimentation was tried but proved injurious to the child. A wet-nurse was then procured, after which the child recovered and is now doing well. The mother regained her strength slowly but fully ; she suffered for a time, however, from looseness of the bowels and indigestion.

In the cicatrix at the line of incision and at the point of insertion of the sutures a remarkable degree of pigmentation took place. Dr. Hartman stated that the entire cicatrix became deeply pigmented—in fact, almost black. The patient herself declared that this pigmentation did not begin to appear until after labor (?) It reached the maximum degree of color after delivery, from which time it began to fade, and at the end of nine weeks had almost disappeared, leaving only a light brown cicatrix.

The tumor was a dermoid cyst with the usual characteristics of such tumors. At the time of removal it was about the size of a child's head at term ; it now appears considerably smaller on account of the shrinking of the cyst wall in the alcohol. The outer surface is smooth, free from adhesions, but uneven ; in some places thin, in others consisting of hard, nodular tumors from a quarter of an inch to an inch in diameter. One portion of it forms a solid mass the size of a small hen's egg, which consists of whitish solid tissue and includes a cyst, the size of a walnut, densely packed with brownish hair. On the inner wall of the larger cyst, which is smooth in its upper portion, may be seen, down near the large tumor, a number of smaller cysts from the size of a pea to that of a hazelnut. In some places the cyst wall is quite thin and transparent, indicating the liability of rupture upon manipulation or by pressure during delivery.

*Remarks.*—Ovarian tumors, which are at all times a source of danger, are still more so when complicating pregnancy, as the two conditions, when in combination, mutually influence each other, to the detriment of both mother and child. The ovarian tumor is subject to acceleration of growth, to more rapid development, during pregnancy. The gravid uterus is liable to cause torsion of the pedicle by changing the form and position of the latter, or by circulatory disturbances in the pedicle, resulting in gangrene or perforation of the cyst. When situated in the pelvis minor, an ovarian tumor is especially liable to become an obstacle to the delivery of the child, and to cause difficult and consequently dangerous labor, which may result fatally to both mother and child.

In discussing the measures for the prevention of these dangers, we will first consider the fate of the mother and child when pregnancy is left to run its course. The dangers to the mother, as gathered from the statistics, are the following : Litzmann has collected fifty-four cases, with twenty-four maternal deaths ; Jetter, two hundred and fifteen deliveries in one hundred and sixty-five mothers, with sixty-four deaths ; Playfair, fifty-seven deliveries, with twenty-three deaths ; Braxton Hicks, six deliveries, with no deaths ; Rogers, five deliveries, with no deaths ; Spencer Wells, eleven deliveries, with one death ; Fritsch, four deliveries, with one death. In all three hundred and fifty-five deliveries are reported, with one hundred and thirteen maternal deaths, or a maternal mortality of about thirty-two per cent.

The mortality to the children from either abortion or premature labor, according to Engstrom, is much greater. In a series of two hundred and sixteen cases a mortality is reported of forty-eight per cent.

The proliferating cystoma is the form of cyst most commonly observed. They are frequently located outside of the small pelvis, and are often overlooked during pregnancy. They rapidly increase in size, and may cause over-distention of the abdomen and severe pressure symptoms from the organs of the abdomen and thorax, necessitating speedy relief. In such cases the treatment by puncture comes in question. As these cysts are located outside of the small pelvis, they are not liable to prove a serious impediment to delivery. Thus it would seem that small dermoid cysts located in the pelvis minor constitute the gravest complication of ovarian tumors with pregnancy.

Dermoid cysts are common. Jetter found thirty-seven dermoid cysts in one hundred and sixty-five cases. They are often small and thus remain in the pelvis, are easily diagnosed by vaginal examination, and therefore, as Olshausen states, are seldom overlooked. These are the tumors which most frequently prove a serious difficulty at the time of delivery, when immovably incarcerated in the pelvis minor.

Puncture of the dermoid cyst is dangerous, as its contents are more poisonous than that of most of the other ovarian tumors ; but puncture becomes unavoidable at the time of delivery when the cyst cannot be pushed out of the way up into the abdominal cavity. The usual location of dermoid cysts in the pelvis minor makes liable the occurrence of spontaneous rupture during delivery, with consequent septic peritonitis resulting partially from infection from the contents of the cyst, and partially from mixed infection through the puerperal wounds.

*Treatment.*—While, outside of pregnancy, prompt extirpation of an ovarian tumor is always indicated, widely different measures have been advocated for the treatment of ovarian tumor when complicated with pregnancy.

1. Induction of abortion and premature labor has been recommended by Barnes, but in most cases this sacrifices the child and is not without danger to the mother. In five cases cited by Olshausen two mothers died. As ovariotomy necessarily must follow, this method of treatment exposes the mother to the dangers of two serious operations.

2. Puncture of the cyst to relieve the symptoms and so permit natural labor to be undisturbed. This procedure, like the preceding one, is of course only temporary and resorted to with a view of awaiting the earliest opportunity for ovariotomy. Puncture of the ovarian tumor may relieve the dyspnœa and prevent abortion. It is not more dangerous in pregnancy than under ordinary circumstances, but the puncture of ovarian tumors in general is attended by a mortality of nineteen per cent. Cohn states that one out of every six ovarian cysts is malignant; therefore puncture might cause rapid diffusion of the malignant tumor in the peritoneal cavity—malignant peritonitis. The more rapid growth of ovarian tumors during pregnancy is apt to cause refilling of the cyst after puncture, and thus necessitate repeated punctures, which, of course, will increase the danger to the mother. Cohnstein states that of six mothers in whom puncture had to be repeated three or more times during pregnancy, five, or eighty-three per cent., died within a short time after delivery from exhaustion. Puncture does not predispose to the interruption of pregnancy in more than eighteen per cent. of the cases.

The difficulty in differential diagnosis between an ovarian tumor and the gravid uterus is apt to lead to puncture of the latter. Olshausen states that in seven cases the uterus was mistaken for an ovarian tumor and punctured. The operator then made a Cæsarean section, sutured the uterus, and closed up the abdomen. This was done in five cases with success; in two cases the puncture terminated fatally.

3. During the last few years a third method of treatment of ovarian tumors during pregnancy has come into the field, namely, ovariotomy during pregnancy. This operation is comparatively new, as in 1877, according to Olshausen, only fourteen cases were on record. In the next year over forty cases were on record, and now this method of treatment bids fair to become a regularly established procedure. Although ovariotomy in the pregnant woman was at first performed with a good deal of apprehension, it has been seen from the very beginning, that the dangers were highly overrated, and that the mortality for mother and child has been decreased by this operation far beyond our expectations. In 1886 Olshausen collected eighty-two cases with only eight deaths, but he points out that individual operators had a much lower mortality, as out of thirty-six cases operated upon by Lawson Tait, Spencer Wells, and Schroeder, only one mother died.

Engström, in 1890, reported a series of forty-eight cases with only two maternal deaths, or a mortality of four and two-tenths per cent, as follows: Schroeder, twelve cases, no deaths; Lawson Tait, eleven cases, one death; Spencer Wells, ten cases, one death; Olshausen, eight cases, no deaths; and Engström, seven cases with no deaths.

I consider the mortality of the operation to-day to be below five per cent. therefore, ovariotomy during pregnancy is not any more dangerous than this operation in the non-pregnant condition.

The fate of the child is influenced by this operation to a like favorable degree. According to Olshausen, abortion follows ovariotomy in only twenty per cent. of the cases; hence eighty per cent. of the children were born at full term. When we compare this with the mortality to the children of forty-eight per cent. with non-interference, we see that by ovariotomy twenty-eight per cent. of the children are saved.

It is generally thought, and probably it is true, that the earlier in pregnancy an ovariotomy is performed the more favorable is the result. Wilson states that ovariotomy becomes less favorable after the fifth month, because, as Schroeder has pointed out, the operation becomes more difficult by shortening of the pedicle, on account of the unfolding and filling in of the broad ligament to which the tumor belongs by the gravid uterus. Late in pregnancy the size of the uterus naturally makes the operation difficult by decreasing the available operating space in the abdominal cavity. This sometimes necessitates the inconvenient lateral operation to gain access to the ovarian tumor. The vascularity of the tumor and pedicle late in pregnancy always increases the difficulty of the operation. But in such cases the facts have proven a surprise to our expectations. Olshausen reports twenty-one cases operated upon after the fourth month, with only two deaths. Pippingsköld reports an operation made after the commencement of labor which resulted successfully. Stratz reports fourteen operations performed by Schroeder, with no maternal deaths and with twelve living children, and formulates the answer to the question whether ovariotomy should always be performed during pregnancy, that it should be done as soon as the diagnosis is made, because:

1. Ovariotomy is inevitable, and its prognosis is not aggravated by the presence of pregnancy.
2. Delivery in childbed without the tumor has a much better prognosis than when the tumor exists.
3. One out of six tumors is malignant, contraindicating puncture.
4. Prognosis for children is much better.

He formulates the following conclusion: "The complication of ovarian tumor with pregnancy indicates ovariotomy."

In the discussion which followed the reading of this paper Weit and Löhlein protested against laying down absolute rules, and suggested that it might be well to individualize. Schroeder, however, fully supported Stratz's recommendation always to operate.

*Final Remarks.*—Small tumors in the pelvis minor are especially dangerous to the child and mother, as has been well illustrated in a case published by Lomer, in which a secondipara, 21

years of age, had an ovarian tumor in the small pelvis the size of a child's head; after rupture of the bag of waters extraction by the foot was tried in vain. Prolapse of the umbilical cord and death of the child resulted, followed next day by version in narcosis, during which the child's head was torn off, and the patient died from collapse in three hours.

In another case, published by Nölting, a small ovarian tumor in the pelvis made delivery difficult in the following way: Forceps were first applied in vain; puncture of the tumor evacuated only a small amount of blood. The child died, and was only extracted after perforation, and still with difficulty, as the tumor came down so far in Douglas' fossa that prolapses of the rectum took place. The patient died after four days of peritonitis. The autopsy showed a double twist of the pedicle, with rupture of the cyst.

Instances of this kind on the one hand, and the low mortality of ovariotomy during pregnancy on the other, would tend to lead to the conclusion that in small ovarian tumors located in the small pelvis and diagnosed during pregnancy immediate ovariotomy is the safest procedure.

BIBLIOGRAPHY.

Olshausen: Die Krankheiten der Ovarien, p. 129.
Stratz: Ueber der complication der Tumoren und Gravidität, Zeitschr. f. Geburts. und Gynäkologie, Band xii., Heft. 2, p. 262.
Engström: Annales de Gynécologie, October and November, 1890.
Cohnstein: Volkmann's Sammlung klinische Vorträge, No. 59.
Lomer: Centralblätt för Gynäkologie, No. 42, 1890.
Nölting: Ibid.

